# A nuclear contortionist: the mitotic migration of *Magnaporthe oryzae* nuclei during plant infection

**DOI:** 10.1080/21501203.2018.1482966

**Published:** 2018-06-12

**Authors:** Mariel A. Pfeifer, Chang Hyun Khang

**Affiliations:** Department of Plant Biology, University of Georgia, Athens, GA30602, USA

**Keywords:** Appressoria, intermediate mitosis, *Magnaporthe oryzae*, mitosis, nuclear envelope, nuclear migration, nuclear plasticity, rice blast, semi-closed mitosis

## Abstract

*Magnaporthe oryzae* is a filamentous fungus, which causes significant destruction to cereal crops worldwide. To infect plant cells, the fungus develops specialised constricted structures such as the penetration peg and the invasive hyphal peg. Live-cell imaging of *M. oryzae* during plant infection reveals that nuclear migration occurs during intermediate mitosis, in which the nuclear envelope neither completely disassembles nor remains entirely intact. Remarkably, in *M. oryzae*, mitotic nuclei show incredible malleability while undergoing confined migration through the constricted penetration and invasive hyphal pegs. Here, we review early events in plant infection, discuss intermediate mitosis, and summarise current knowledge of intermediate mitotic nuclear migration in *M. oryzae.*

## Introduction

*Magnaporthe oryzae*, also known as the rice blast fungus, is a filamentous hemibiotrophic plant pathogen. It is capable of mass destruction to valuable plant crops such as rice and wheat, as well as barley, finger millet, and foxtail millet (Gladieux et al. 2018). In fact, each year *M. oryzae* causes an estimated $66 billion in economic damage to rice crops, destroying enough food to have fed 60 million people (Pennisi 2010). In the field, *M. oryzae* is developing increased resistance to commonly used fungicides (Ribas e Ribas et al. 2016) and, recently, wheat blast emerged in Bangladesh (Islam et al. 2016; Malaker et al. 2016). Understanding cellular processes unique to *M. oryzae* is an important first step in the development of novel and effective methods to control the deadly plant pathogen and ensure global food security.

Proper positioning of the nucleus within eukaryotic cells is vital and relies upon successful nuclear migration into incipient cells (Morris 2000). A full gamut of mitotic forms is possible in eukaryotes, ranging from completely closed to completely open (Heath 1980; Boettcher and Barral 2013). In fungi, nuclear migration into incipient cells occurs before, during, or after mitosis (Gladfelter and Berman 2009). Recent studies reveal that *M. oryzae* undergoes mitosis that is not completely closed or open and that the mitotic nucleus becomes highly deformed while migrating through narrow structures that arise during plant infection (Jones et al. 2016a; Jenkinson et al. 2017).

In this review, we highlight the nuclear dynamics of *M. oryzae* during plant infection with a focus on mitosis and mitotic nuclear migration. We contextualise these cellular processes by discussing the early events in rice blast infection and the range of mitotic programmes documented in fungi. We provide an outlook on what mechanisms of nuclear migration likely exist in *M. oryzae* and discuss whether similar cellular processes are present in other plant pathogens.

## Early events in rice blast infection

Rice blast infection begins when an asexual three-celled conidium attaches to the surface of a rice plant (Hamer et al. 1988). A polarised germ tube develops, and the fungus forms a melanised dome-shaped cell called an appressorium in the presence of the appropriate extracellular physical and chemical signals, for instance, hydrophobicity of the leaf surface (Veneault-Fourrey et al. 2006; Ryder and Talbot 2015). The most apical nucleus of the conidium undergoes mitosis, and one nucleus migrates to the incipient appressorium, followed by autophagy of the conidium (Veneault-Fourrey et al. 2006; Saunders et al. 2010a). It remains unknown if extracellular cues trigger mitosis during appressorium development. Accumulation of turgor pressure in the appressorium in coordination with septin-dependent cytoskeletal rearrangements at the appressorial pore leads to formation of the penetration peg (Howard et al. 1991; Dagdas et al. 2012). The penetration peg is a specialised hypha that physically breaches the leaf cuticle, allowing the fungus to enter rice cells approximately 24 h post-inoculation (Kankanala et al. 2007). Several S-phase checkpoints have been identified, which regulate appressorium development and formation of the penetration peg (Saunders et al. 2010a; Osés-Ruiz et al. 2017). Once inside the first-invaded rice cell, the penetration peg gives rise to the primary hypha (Kankanala et al. 2007). The apical tip of the primary hypha switches from filamentous to depolarised growth causing the apical tip to swell (Shipman et al. 2017). Tip expansion of the primary hypha appears to serve as a size threshold which triggers mitosis in the single nucleus located in the appressorium (Shipman et al. 2017). This nucleus begins mitosis inside the appressorium and undergoes a long-distance migration during presumed anaphase B to its eventual position in the swollen tip of the primary hypha (Jenkinson et al. 2017; Shipman et al. 2017). Subsequently, septation occurs, and the first cell of the bulbous invasive hyphae (IH) is formed (Shipman et al. 2017). Incongruent descriptions of the exact location of mitosis at this infection stage exist in the literature. Two previous reports describe the appressorial nucleus migrating from the appressorium into the primary hypha and then undergoing mitosis, rendering the appressorium anucleate for a time (Veneault-Fourrey et al. 2006; Fernandez et al. 2014). However, later work reports mitosis as most commonly occurring within the appressorium (Jenkinson et al. 2017; Osés-Ruiz et al. 2017; Shipman et al. 2017). The reason for this discrepancy remains unknown.

*M. oryzae* secretes effector proteins during plant infection. Effectors function to dampen plant immune responses and change the metabolism of the plant (Giraldo et al. 2013). The biotrophic interfacial complex (BIC) is a plant-derived membrane-rich structure that lies outside the fungal cytoplasm and is the site at which cytoplasmic effectors accumulate during plant infection (Khang et al. 2010; Giraldo et al. 2013). The BIC first appears at the tip of the primary hypha but then is repositioned to the side of the first bulbous cell (Khang et al. 2010; Shipman et al. 2017). Cytoplasmic effectors are secreted to the BIC via non-conventional secretion and eventually enter plant cells, while apoplastic effectors undergo conventional secretion and remain contained within the extra-invasive hyphal membrane (Giraldo et al. 2013).

To invade neighbouring plant cells, IH appear to seek out pit fields and undergo a morphological shift from polarised to isotropic growth controlled by the Pmk1 mitogen-activated protein kinase (Kankanala et al. 2007; Sakulkoo et al. 2018). IH then form highly constricted IH pegs to cross plant cell walls (Kankanala et al. 2007). The first-invaded rice cells are alive but die when the fungus enters adjacent cells (Kankanala et al. 2007; Jones et al. 2016b) Colonisation of the first-invaded plant cell takes approximately 8–12 h, while in subsequently invaded cells, the fungus only develops for 2–3 h (Kankanala et al. 2007; Jones et al. 2016b). One possible explanation for the difference in colonisation time between first-invaded and subsequently invaded cells is that effectors move cell-to-cell likely through plasmodesmata and prime neighbouring cells for fungal invasion (Kankanala et al. 2007; Khang et al. 2010). Within 4–5 days of initial infection, blast lesions become apparent on the plant tissue (Sakulkoo et al. 2018).

## The mitotic spectrum in fungi

A fundamental property of eukaryotic life is the division of replicated genetic information to new daughter cells through mitosis. Typically, when we think of mitosis, we recall cellular events characteristic of mitosis in plant and animal cells that use completely open mitosis where the nuclear envelope (NE) disassembles during prophase. However, multiple forms of mitosis exist across the eukaryotic domain (Arnone et al. 2013; Sazer et al. 2014; Makarova and Oliferenko 2016). In fungi, it is often assumed that all species rely on completely closed mitosis, yet, closer study of mitotic nuclear dynamics reveal a spectrum of mitotic programmes in fungi (Heath 1980; De Souza and Osmani 2007).

Mitosis is categorised based on the state of the NE during nuclear division (Arnone et al. 2013; Sazer et al. 2014; Makarova and Oliferenko 2016). In completely closed mitosis, the NE remains entirely intact throughout nuclear division. Spindle pole bodies, embedded within the NE, serve as microtubule-organising centres and permit the spindle to form within the nucleus (Arnone et al. 2013). The spindle elongates in coordination with NE expansion, and eventually the condensed chromosomes are separated. Conversely, in completely open mitosis, all components of the NE undergo systematic dismantling during prophase (Arnone et al. 2013). This regulated disassembly of the NE grants the spindle, which is nucleated in the cytoplasm, access to chromosomes which are then separated. The NE is reformed at the end of mitosis, enclosing two daughter nuclei.

Historically, classification of closed or open mitosis was determined by observing the state of the NE using transmission electron microscopy throughout all phases of mitosis in classical model organisms, such as the closed mitosis of *Saccharomyces cerevisiae* (Sazer et al. 2014). As studies of NE dynamics during mitosis expanded beyond model organisms, it became obvious that classifying mitosis as either completely closed or completely open is not always clear, especially in fungi (Heath 1980). The NE of some species persists throughout mitosis but is not fully intact, thus exhibiting characteristics of both a closed and open mitosis (Heath 1980; Sazer et al. 2014). Mitosis that is not completely closed nor completely open is called intermediate mitosis (De Souza and Osmani 2007; Arnone et al. 2013). Intermediate mitosis involves changes to the structure and integrity of the NE, which leads to a significant loss of compartmentalisation between the nucleus and the cytoplasm. Several terms exist to describe forms of intermediate mitosis, for instance, semi-open in *Aspergillus nidulans* (Lin et al. 2015), modified-open in *Ustilago maydis* (Straube et al. 2005), and partially open as a collective term for all forms of intermediate mitosis (Arnone et al. 2013; Sazer et al. 2014). Generally, mechanisms of intermediate mitosis in fungi can be grouped into two broad categories: tearing of the NE or altering the composition of a largely intact NE to enhance permeability. Fungi such as *U. maydis* and *Schizosaccharomyces japonicus* experience NE tearing during mitosis (Straube et al. 2005; Aoki et al. 2011; Yam et al. 2011). In *A. nidulans*, a subset of proteins found within nuclear pore complexes (NPCs) disperse into the cytoplasm during mitosis (De Souza et al. 2004; Osmani et al. 2006).

## Intermediate mitosis in *Magnaporthe oryzae*

**Figure 1.**
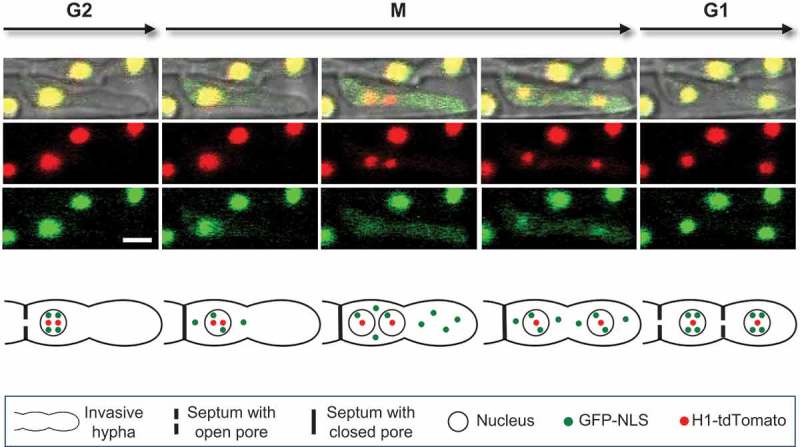
Time-lapse confocal fluorescence images and schematic diagram of intermediate mitosis in an invasive hypha of *M. oryzae* within the first-invaded rice cell. This strain expresses histone H1-tdTomato and GFP-NLS. The top panel shows five sequential fluorescence pattern stages in both merged bright-field and fluorescence (top), red fluorescence alone (middle), and green fluorescence alone (bottom). The interphase nucleus in G2 appears yellow due to colocalization of H1-tdTomato (red) and GFP-NLS (green) within the nucleus. H1-tdTomato remains associated with DNA throughout the cell cycle. During the early stages of mitosis (M), GFP-NLS spills into the cytoplasm, indicating a change to the integrity of the nuclear envelope. GFP-NLS is contained within the dividing cell by presumed closure of septal pores. Following mitosis, GFP-NLS is reimported back into the nucleus and the nucleus again becomes yellow during interphase (G1). The bottom panel presents a schematic summary of these cellular events. Bar = 5 μm. This figure is modified from Jones et al. (2016a). To view this figure in color, please see the online version of this journal.

Mitosis in *M. oryzae* bears a hallmark of intermediate mitosis, the dramatic loss of compartmentalisation between the nucleus and the cytoplasm. During mitosis, cytoplasmic ZsGreen signal equalises between the cytoplasm and the nucleus (Bourett et al. 2002), and the import of tubulin into the nucleus increases dramatically after mitotic onset (Czymmek et al. 2005). Following the dynamics of green fluorescent protein fused with nuclear localisation signal (GFP-NLS) during plant infection provides further evidence of intermediate mitosis (Jones et al. 2016a; Jenkinson et al. 2017). GFP-NLS signal remains within the nucleus during interphase in the appressorium and IH. However, at the start of mitosis, GFP-NLS signal becomes cytoplasmic with subsequent reimportation of GFP-NLS once mitosis is complete (Figure 1). Together, these reports show that mitosis in *M. oryzae* is not completely closed where the NE would be expected to function as an intact barrier to prevent mixing of nuclear and cytoplasmic proteins. Possible explanations for this observed loss of compartmentalisation include (1) the NE of *M. oryzae* tears or (2) the NE persists but loses integrity during mitosis. Differentiating between these two possibilities requires following the dynamics of the NE throughout mitosis.

Staining the outer nuclear membrane using the lipophilic dye 3,3ʹ-dihexyloxacarbocyanine iodide showed that the outer nuclear membrane remains intact during mitosis in the germ tube at the appressorium development stage of infection (Saunders et al. 2010b). Additional studies of the NE at the same infection stage reveal that the core nucleoporin (Nup), Nup84, localises to the polar edges of the dividing nucleus (Pfeifer and Khang, unpublished data). The persistence of Nup84 at the NE throughout mitosis confirms that portions of the NE do remain intact during nuclear division. Additionally, the polar localisation of Nup84 in *M. oryzae* is similar to other fungi known to use intermediate mitosis, including *S. japonicus* (Aoki et al. 2011; Yam et al. 2011) and *A. nidulans* (Osmani et al. 2006). While these results confirm distinct components of the NE remain intact during mitosis, the details of the mechanism responsible for intermediate mitosis remain to be discovered in *M. oryzae*.

Interestingly, *M. oryzae* shares many mitotic similarities with *A. nidulans*, a model organism with well-studied mitotic dynamics. The NE of both species remains intact throughout mitosis (Robinow and Caten 1969; Saunders et al. 2010b) while the permeability of the NE increases during mitosis (Suelmann et al. 1997; Bourett et al. 2002; Ovechkina et al. 2003; De Souza et al. 2004; Czymmek et al. 2005; Osmani et al. 2006; Jones et al. 2016a; Shipman et al. 2017). Furthermore, *A. nidulans* and *M. oryzae* show loss of compartmentalisation between the nucleus and cytoplasm with a coordinated closure of septal pores during mitosis (Ovechkina et al. 2003; De Souza et al. 2004; Osmani et al. 2006; Shen et al. 2014; Jones et al. 2016a). In *M. oryzae*, cytoplasmic GFP-NLS remains contained within the dividing cell during mitosis, which suggests cell cycle regulation of septal pores (Jones et al. 2016a). A similar case is observed in *A. nidulans* where the NIMA kinase coordinates septal pore opening and closing to be out of sync with the dispersal of Nups from NPCs during prophase (Shen et al. 2014). This regulated closure of septal pores likely prevents diffusion of mitotic kinases throughout neighbouring cells thereby preventing precocious mitoses which could be detrimental to the fungus (Shen et al. 2014). Future study of the NIMA homolog will reveal if similar regulation of septal pores exists in *M. oryzae*.

Although mounting evidence strongly suggests that *M. oryzae* uses a form of intermediate mitosis, details about the process are lacking. Data describing the dynamics of all components of the NE throughout mitosis is needed to fully characterise the form of intermediate mitosis used by *M. oryzae*. To date, only the localisation of the outer nuclear membrane and Nup84 has been studied during mitosis at one stage of rice blast infection, appressorium development. An important direction for future research is to visualise the inner nuclear membrane using a combination of confocal and transmission electron microscopy throughout mitosis to fully describe whether this component of the NE remains intact during mitosis in *M. oryzae*. Given the mitotic similarities to *A. nidulans*, we hypothesise that a key feature of mitosis in *M. oryzae* is dispersal of peripheral Nups during prophase while other NE components (the outer and inner nuclear membranes along with core Nups) remain intact during mitosis. Demonstrating that core Nups remain associated with the NE during mitosis while peripheral Nups localise to the cytoplasm will implicate a mechanism akin to *A. nidulans* (Osmani et al. 2006).

Although we hypothesise *M. oryzae* shares key mitotic features with *A. nidulans*, it is important to note that nuclear distribution differs between the two species. *M. oryzae* is typically a mononuclear species, with only one nucleus per cell. *A. nidulans*, however, has multiple nuclei within one common cytoplasm. Since *A. nidulans* is syncytial, the persistence of the NE may help prevent spindle microtubules from interacting with the chromosomes of a nearby dividing nucleus in an adulterous manner (De Souza and Osmani 2007). Why then does *M. oryzae* use intermediate mitosis? The variety of mitotic programmes present in fungi suggests that each type of mitosis conveys advantages and disadvantages to the organism (Boettcher and Barral 2013). We speculate that intermediate mitosis in *M. oryzae* confers a to-be-determined advantage, perhaps to permit more efficient plant infection. However, research in this area is very much in its nascence. Future work in *M. oryzae* may better address what advantages intermediate mitosis brings to fungi during plant infection.

## Nuclear constrictions during plant infection

*M. oryzae*’s IH are highly plastic as they grow inside plant cells. For instance, during cell-to-cell movement as IH cross from the first-invaded cell to adjacent cells via the narrow IH peg, IH constrict from an initial average diameter of 5 to 0.5 μm (Kankanala et al. 2007; Sakulkoo et al. 2018). Live-cell imaging of cell-to-cell movement revealed that hyphae developing in the newly invaded neighbour cell must grow significantly before becoming nucleated (Kankanala et al. 2007). These results suggested that nucleation of the incipient fungal hypha depends upon mitosis and the successful delivery of the daughter nucleus across the IH peg. Until recently, the details of this process remained enigmatic, largely due to the technical challenges of capturing rapid nuclear migration through the IH peg using time-lapse imaging.

An elegant combination of GFP-NLS and histone H1-tdTomato fluorescent reporter proteins was utilised to study nuclear dynamics during plant infection. GFP-NLS spill from the nucleus into the cytoplasm indicates entrance into prophase and that the nucleus will divide within a few minutes, while histone H1-tdTomato remains associated with DNA throughout the cell cycle (Jones et al. 2016a; Jenkinson et al. 2017). This combination of fluorescent proteins provided crucial temporal and spatial cues to time-lapse image nuclear migration events inside the rice plant without causing phototoxicity to divide fungal cells (Jones et al. 2016a; Jenkinson et al. 2017). Using GFP-NLS as an indicator for mitosis demonstrates that nuclear migration occurs during intermediate mitosis. That is, GFP-NLS disperses from the nucleus before migration occurs and is not fully reimported back until after nuclear migration (Jones et al. 2016a; Jenkinson et al. 2017).

**Figure 2.**
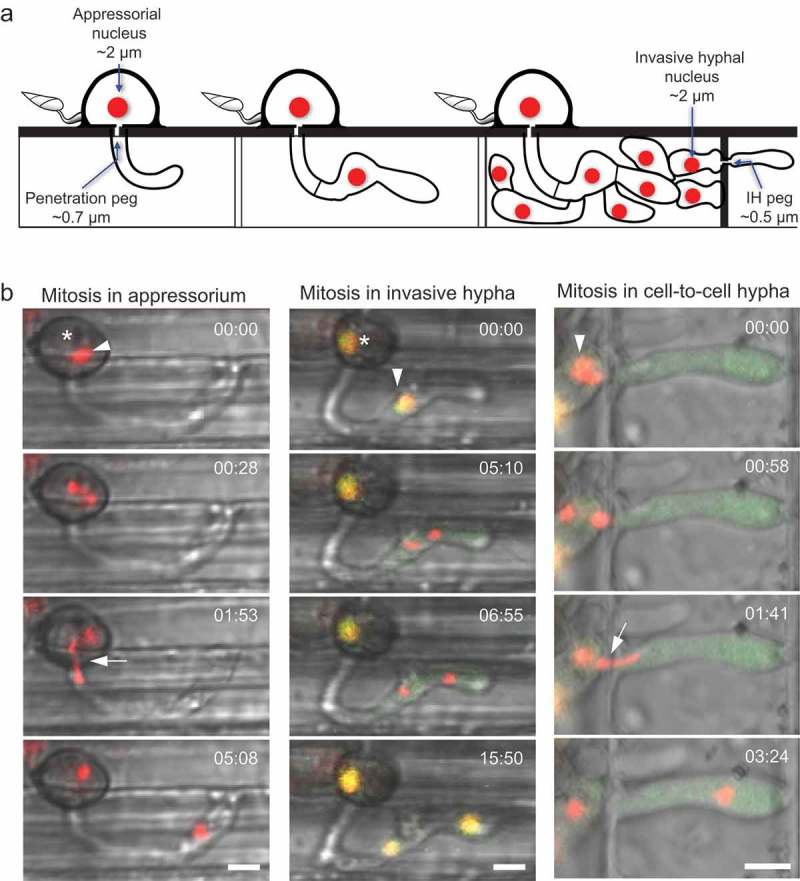
Mitotic migration of *M. oryzae* nuclei during early rice blast infection. (a) Schematic diagram summarizing key cellular structures and mononuclear positioning during plant invasion. The nucleus in the appressorium (interphase diameter of ~2 μm) must traverse the constricted penetration peg (diameter of ~0.7 μm) for final receipt in the incipient primary hypha. Once inside the first-invaded cell, the primary hypha becomes bulbous to form invasive hyphae (IH). To move into adjacent rice cells, IH seek out pit fields and develop a constricted IH peg (diameter of ~0.5 μm). (b) A time-lapse series of nuclear dynamics at three distinct stages of early rice blast infection. Asterisks denote the appressorium, arrowheads label a nucleus about to undergo mitotic nuclear migration, and arrows highlight extreme nuclear morphology during confined nuclear migration through peg structures. (Left: merge of bright-field and H1-tdTomato.) Mitosis begins in the appressorium, and the daughter nucleus becomes highly constricted and elongated during confined mitotic nuclear migration through the penetration peg. The original nucleus remains located in the appressorium throughout this event. GFP-NLS dynamics (data not shown) confirms nuclear migration occurs during intermediate mitosis at this infection stage (Jenkinson et al. 2017). (Middle panel: merge of bright-field, GFP-NLS, and H1-tdTomato.) During mitosis in the invasive hypha, the interphase nucleus appears yellow due to colocalisation of H1-tdTomato and GFP-NLS in the nucleus. After onset of mitosis, GFP-NLS disperses into the cytoplasm, and the nucleus undergoes an unconfined nuclear migration. Following receipt of the nucleus into the new invasive hypha cell, mitosis ends, and GFP-NLS is fully reimported back into the nucleus. (Right panel: merge of bright-field, GFP-NLS, and H1-tdTomato.) Here, confined nuclear migration through the IH peg occurs. In early mitosis, the sister chromatids separate and during presumed anaphase B, a single daughter nucleus undergoes confined nuclear migration through the constricted IH peg. The daughter nucleus again becomes spherical and continues to migrate to the tip of the IH in the second-invaded cell prior to GFP-NLS reimport into the nucleus. Times are shown in minutes:seconds. Bars = 5 μm. This figure is modified from Jones et al. (2016a) and Jenkinson et al. (2017).

These studies further revealed several important findings about nuclear migration during plant infection (Figure 2 and full videos are available on youtube.com by searching Khang Lab at UGA Rice Blast). Firstly, the nucleus adopts an extremely constricted morphology as it migrates through the IH peg. One nucleus (interphase diameter of ~2 μm) was observed to stretch to over 5 μm in length while moving through the IH peg (Jones et al. 2016a). After squeezing through the IH peg, the nucleus assumed its typical spherical shape and continued to migrate a total distance of 16.9 μm from the site of chromosome separation to the now nucleated fungal hyphal cell located in the second-invaded rice cell (Jones et al. 2016a). This remarkable nuclear morphology was also observed at an earlier stage of plant infection, when the appressorial nucleus moved through the penetration peg (diameter of .7 μm) during mitosis (Jenkinson et al. 2017; Shipman et al. 2017). Here, the appressorial nucleus became highly constricted and elongated with a maximum length of 13 μm reported (Jenkinson et al. 2017). At both stages of infection, the migrating nucleus appeared to be tethered to the mother nucleus (Jones et al. 2016a; Jenkinson et al. 2017). Notably, only the migrating nucleus could enter and cross the penetration or IH peg. Together, these observations suggest that *M. oryzae* possesses a cellular mechanism responsible for the confined migration of the mitotic nucleus through narrow pegs arising during plant infection.

The ability of *M. oryzae*’s nuclei to withstand such extreme constrictions during plant infection is certainly captivating. Fungal nuclei are known to be flexible, largely attributed to the fact that fungi lack true lamin proteins (Steinberg et al. 2012; Ciska and Moreno Díaz De La 2014). The nuclei of fungi such as *U. maydis* (Straube et al. 2005), *Neurospora crassa* (Roca et al. 2010), and *Candida albicans* (Finley and Berman 2005) all display some degree of elongation during migration. However, to our knowledge, the morphology adopted by the nuclei of *M. oryzae* during confined migration through the penetration or IH peg is the most drastic morphology to be reported.

## Nuclear migration during plant infection

Nuclear migration in many fungi requires the coordination of microtubules, the motor protein cytoplasmic dynein, and additional microtubule-associated proteins; for extensive reviews of this topic, see Gladfelter and Berman (2009), Roberts and Gladfelter (2016), and Xiang (2018). For example, in *S. cerevisiae*, astral microtubules are nucleated at the spindle pole body and rely on dynamic instability to search the cell cortex of the bud to locate Num1, a cortical dynein-interacting protein (Carminati and Stearns 1997; Heil-Chapdelaine et al. 2000; Farkasovsky and Küntzel 2001). Once the plus-ends of astral microtubules bind to Num1, they slide against the cell cortex in a dynein-mediated manner which moves the spindle and eventually the daughter nucleus into the newly formed bud (Heil-Chapdelaine et al. 2000). Functional homologs of Num1 are also required for proper nuclear migration in multinucleate fungi, including *A. nidulans* (Veith et al. 2005) and *Ashbya gossypii* (Grava et al. 2011). In *M. oryzae*, a Num1 homolog, MoAND1, has been characterised (Jeon et al. 2014). Without MoAND1, nuclear positioning in vegetative hyphae and conidia is affected (Jeon et al. 2014). Importantly, ΔMoand1 shows a reduced ability to initially invade rice cells, suggesting that a cortical dynein anchor is necessary for the fungus to penetrate plants.

A recent study of a class myosin-II motor, Momyo2, in *M. oryzae* showed that disruption of Momyo2 resulted in aberration in nuclear distribution (Guo et al. 2017). Like the ΔMoAND1, the ΔMomyo2 strain shows reduced ability to penetrate into plants (Guo et al. 2017). In other fungi, class-II myosins in cooperation with actin play important roles in cytokinesis and septation (Takeshita 2016). Future research investigating the functions of microtubules and actin along with associated motor proteins will yield valuable information about *M. oryzae*’s mechanism of nuclear migration during plant infection.

## Outlook and conclusion

We now know that *M. oryzae* nuclei become extremely constricted while migrating through the confined channels of the penetration or IH peg and that nuclear migration occurs during intermediate mitosis. Does the NE regularly rupture during mitotic migration? Is NE rupture more frequent as the nucleus migrates through the pegs? What are the motor and accessory proteins needed for successful nuclear migration during plant infection? The answers to these intriguing questions remain to be discovered.

Interestingly, other fungi also form confined structures during plant infection. For example, *Colletotrichum* spp. form an appressorium-derived penetration peg to initially enter their host cells (Mendgen et al. 1996; Nesher et al. 2008; De Silva et al. 2017). *Fusarium graminearum* forms intracellular hyphae that exhibit apparent constriction during cell-to-cell movement (Jansen et al. 2005). Although it is currently unknown whether *F. graminearum* uses intermediate mitotic nuclear migrations during infection, some other *Fusarium* spp., such as *F. oxysporum* and *F. verticillioides*, show signs of intermediate mitosis (Bourett et al. 2002; De Souza and Osmani 2007). Future work in filamentous fungi including *M. oryzae* will provide evidence needed to draw broader conclusions regarding conserved mechanisms of mitotic nuclear migration during plant infection.

As technology advances to allow studies of cellular processes at single-cell resolution, an important aim will be to discover how intracellular fungal pathogens position nuclei during plant infection. *M. oryzae* represents one of the most significant threats to global food production, and resistance to fungicides such as azoles is increasing (Ribas e Ribas et al. 2016). Investigating nuclear migration in *M. oryzae* during plant infection will likely identify fungal-specific cellular targets to halt nuclear migration and thereby prevent infection progression. Insight into these fascinating cellular mechanisms could aid in the development of new fungicides to control the deadly rice blast fungus and other fungal plant pathogens.
